# Sociodemographic and health profile of asylum-seekers in Rio de Janeiro, 2016–2017

**DOI:** 10.11606/s1518-8787.2022056003956

**Published:** 2022-04-13

**Authors:** João Roberto Cavalcante, Raquel Proença, Ignacio Cano, Anete Trajman, Eduardo Faerstein

**Affiliations:** I Universidade do Estado do Rio de Janeiro Instituto de Medicina Social Rio de Janeiro RJ Brasil Universidade do Estado do Rio de Janeiro. Instituto de Medicina Social. Rio de Janeiro, RJ, Brasil; II Universidade do Estado do Rio de Janeiro Centro de Ciências Sociais Rio de Janeiro RJ Brasil Universidade do Estado do Rio de Janeiro. Centro de Ciências Sociais. Rio de Janeiro, RJ, Brasil

**Keywords:** Refugees, Population Characteristics, Health Profile, Global Health

## Abstract

**OBJECTIVE:**

To analyze the sociodemographic profile and self-reported health conditions of asylum-seekers in Rio de Janeiro.

**METHODS:**

A cross-sectional study of secondary data, collected from asylum claims forms completed in 2016 and 2017, at Cáritas Arquidiocesana do Rio de Janeiro (Cáritas-RJ). Descriptive analyses were performed and absolute and relative frequencies and 95% confidence intervals were calculated.

**RESULTS:**

Claims completed by 818 asylum-seekers from 49 different countries were identified, of whom 126 (20.3%) were stateless, 510 (62.7%) were male, 797 (97.4%) were adults, with a mean age of 30.5 years, 551 (73.5%) were single, 340 (44.1%) had higher education, and 27 (4.0%) were unemployed in their country of origin before coming to Brazil. Fear of persecution for political opinion, violation of human rights, and risk of torture stood out among the reasons stated for requesting asylum. To reach Brazil, 629 (80.5%) traveled only by plane. Regarding health conditions, 216 (29.0%) reported having some symptom, disease or health problem, the most frequent being pain, vision problems, infectious diseases (including HIV/AIDS), and hypertension. Only 15 individuals (2.2%) reported being in some medical or psychological treatment; 42 (6.0%) reported visual impairments, 14 (2.0%) reported physical impairments and 4 (0.6%) hearing impairments.

**CONCLUSIONS:**

Unlike other countries, where forced migrants with a low level of education enter clandestinely by sea or land, asylum-seekers residing in Rio de Janeiro between 2016 and 2017 were mostly adults with higher education who migrated using air transport. They had primary care-sensitive health conditions that could be treated via access to public primary health care services.

## INTRODUCTION

Forced migrations are not a recent historical phenomenon, on the contrary, they date back to prehistory, when about two million years ago our ancestors moved from Africa to other regions of the planet, mainly due to climate issues and the scarcity of food resources^1^. More recently, other forced migrations occurred for different reasons, such as conflicts and wars, poverty and hunger, epidemics and the search for access to health services^2^.

Forced migrations fall into the following subcategories: refugees, asylum-seekers and internally displaced persons. Refugees are individuals who have received protection status from the country to which they migrated, because they have a well-founded fear of being persecuted due to race, religion, nationality, social group, political opinions or a situation of serious and generalized violation of human rights^3^, are outside the country of their nationality, and are unable or, owing to this fear, are unwilling to avail themselves of the protection of that country^4^. Asylum-seekers are individuals who have already carried out international migration and intend to be admitted to the country of destination as refugees and are awaiting a decision on their asylum claim under the relevant international and national instruments^5^. Internally displaced persons are individuals with migration reasons similar to those of refugees and asylum-seekers, but who have not crossed an international border^6^.

The largest wave of forced migrations since the Second World War is currently taking place in the world. According to the United Nations High Commissioner for Refugees (UNHCR), in 2019 there were 79.5 million forced migrants in the world (1% of the world population): 26 million refugees, 4.2 million asylum-seekers, 45.7 million internally displaced people, and 3.6 million Venezuelans displaced abroad^6^.

In Brazil, in 2019, there were 43,000 individuals with recognized refugee status in the previous 10 years, coming from 90 different countries, of whom 90% were from Venezuela^7^. That year there were 82,500 new asylum claims. The National Committee for Refugees (CONARE) recognized 38,000 Venezuelans as refugees between December 2019 and April 2020 (five months), but thousands of requests made before and after this period are still pending and accumulated awaiting decision^6^.

Migration can have a direct negative impact on the individuals’ health conditions. Diseases and conditions such as tuberculosis, syphilis, hypertension, diabetes mellitus, obesity, depression, anxiety, post-traumatic stress disorder, sequelae of torture, mutilation (including female genital mutilation) and sexual violence are frequent in this population^8,9^. The main difficulties faced after arriving in the country of destination include precarious work, misunderstanding of its culture, communication difficulties, and difficulties in accessing health care^8^. Access to health services and the guarantee of housing, work and income are examples of public policies that can mitigate the adverse effects of post-migration experiences^10^.

Brazil has been sought as a welcoming country by an increasing number of forced migrants. Little is known about the profile and health of this population. In Rio de Janeiro, *Cáritas Arquidiocesana* (Cáritas-RJ) provides assistance to this population, for example, assisting them in dealing with asylum claims and facilitating their access to the Brazilian Unified Health System (SUS). Cáritas-RJ is an organization linked to the Catholic Church, created in 1976, when the then Archbishop of Rio de Janeiro, Dom Eugênio Sales, began a pioneering work of assistance to politically persecuted people in dictatorships who arrived in Rio de Janeiro from neighboring countries, such as Argentina, Chile and Uruguay^11^.

Cáritas-RJ continues to welcome hundreds of refugees and asylum-seekers every year and keeps the asylum claims applications on paper, which contain various data on this population^11^. To the best of our knowledge, studies based on these data have not been conducted. This study aimed to analyze the sociodemographic profile and self-reported health conditions of asylum-seekers in Rio de Janeiro attended to at this institution.

## METHODS

A cross-sectional study was carried out with retrospective secondary data collection, whose population consisted of individuals who filled out the CONARE asylum claim application in Cáritas-RJ in 2016 and 2017, when the form started to contain health information. Additionally, information was collected from interviews carried out by the Cáritas’s team of social workers.

At Cáritas-RJ, asylum-seekers had the option of answering the application in Portuguese, Spanish, English and French. When the applicant did not master any of these four languages, being a speaker, for example, of Kikongo, Lingala, Arabic, etc., interpreters helped in completing the application. The study period applications had 26 pages with 103 questions and their data were extracted by 12 typists between April 2018 and December 2019.

A form was built for study data extraction using EpiData 4.2.0.0 software. The 12 typists were trained to collect data from the asylum claims applications and handle the EpiDATA. Three pre-tests of the form were performed by the 12 typists, who extracted data from a sample of 20 forms, in order to compare and homogenize the typings and make adjustments on the form questions. The claims were classified by language before the beginning of the collection, so that each typist collected the claims in the languages in which they were fluent. Weekly, two supervisors checked about 10% of the forms typed in order to detect and correct possible errors.

Descriptive analyses of categorical variables were performed and absolute and relative frequencies were calculated with their respective 95% confidence intervals (95%CI). The open answers variables, that is, those that were not multiple choice, were presented with the most frequent answers. These same analyses were repeated separately for asylum-seekers from the five most frequent countries of origin. Missing data were not included in the frequency calculations. A thematic map was also prepared with the number of asylum claims by country of origin. Data analyses were performed using the R 3.4.2 software and the thematic map was created using the QGIS 3.14.16 software.

The project was approved by the research ethics committee of the Institute of Social Medicine of the State University of Rio de Janeiro (IMS/UERJ) under protocol 2,437,258.

## RESULTS

We identified and included 818 asylum claims referring to the study period, of which 529 (64.7%) were from 2016 and 289 (35.3%) from 2017. Of these, only 11 (1.3%) had the asylum claim accepted until the time of collection (Table 1). Forty-nine (49) countries of birth identified. The 12 countries that had more than 10 asylum claims were: Angola (231 requests, 28.2% of the total), Democratic Republic of Congo (153, 18.7%), Venezuela (133, 16.2%), Cuba (69, 8.4%), Syria (34, 4.1%), Guinea-Bissau (20, 2.4%), Guinea (18, 2.2%), Pakistan (16, 1.9%), Colombia (15, 1.8%), Sierra Leone (15, 1.8%), Benin (12, 1.4%) and Morocco (12, 1.4%) (Figure).

**Table 1:** Sociodemographic profile of asylum-seekers assisted at Cáritas Arquidiocesana do Rio de Janeiro, between 2016 and 2017.

Sociodemographic characteristics	Answers	n	%	95%CI
Year of filling out the form (n = 818)	2016	529	64.7	61.2–67.9
	2017	289	35.3	32.0–38.7
Request (n = 818) *status*	Pending	758	92.7	90.6–94.3
	Accepted	11	1.3	0.7–2.4
	Refused	1	0.1	0.0–0.7
	Uninformed	48	5.9	4.4–7.7
Sex (n = 818)	Male	510	62.4	58.9–68.6
	Female	308	37.6	34.3–41.0
Age range in years (n = 818)	Under 18	21	2.6	1.6–3.9
	18 to 29 years	427	52.2	48.7–55.6
	30 to 44 years	315	38.5	35.1–41.9
	45 to 59 years	52	6.4	4.8–8.3
	60 years or older	3	0.4	0.0–1.1
Form language (n = 818)	Portuguese	292	35.7	32.4–39.1
	Spanish	233	28.5	25.4–31.7
	French	206	25.2	22.2–28.3
	English	87	10.6	8.6–13.0
Mother tongue (n = 737)	Spanish	216	29.3	26.0–32.7
	Portuguese	185	25.1	22.0–28.4
	Lingala	83	11.3	9.1–13.8
	Kikongo	41	5.6	4.0–7.5
	Arabic	37	5.0	3.6–6.9
	Others	175	23.7	20.7–27.0
Second language (n = 471)	English	105	22.3	18.6–26.3
	French	99	21.0	17.4–25.0
	Portuguese	91	19.3	15.9–23.2
	Lingala	46	9.8	7.3–12.8
	Kikongo	37	7.9	5.6–10.7
	Others	93	19.7	16.3–23.6
Marital status (n=775)	Single	551	71.1	67.7–74.2
	Married	199	25.7	22.6–28.9
	Divorced	11	1.4	0.7–2.6
	Separated	5	0.6	0.2–1.5
	Widowed	4	0.5	0.1–1.4
	Common-law marriage	2	0.3	0.0–1.0
	Free union	3	0.4	0.1–1.2
Education (n = 771)	Higher	340	44.1	40.5–47.7
	Secondary	312	40.5	36.9–44.0
	Primary	119	15.4	12.9–18.2
Sector of occupation in the country of origin (n = 675)	Services	348	51.5	47.7–55.4
	Business	99	15.6	12.1–17.6
	Industry	65	9.6	7.5–12.1
	Agrarian and livestock	4	0.6	0.1–1.6
	Unemployed	27	4.0	2.7–5.8
	Others	132	19.5	16.6–22.7
Religion (n=641)	Christianity	515	80.3	77.0–83.3
	Islam	71	11.1	8.8–13.8
	Without religion	25	3.9	2.6–5.7
	Atheist	3	0.5	0.1–1.4
	Others	27	4.2	2.8–6.1
Christian Denominations (n = 515)	Catholics	223	43.3	38.9–47.7
	Pentecostals	76	14.8	11.8–18.1
	Jehovah’s Witnesses	21	4.1	2.6–6.2
	7th Day Adventists	8	1.6	0.7–3.1
	Protestants	6	1.2	0.4–2.6
	Christians – unspecified	181	35.1	31.0–39.4
Ethnicity (n = 396)	Bakongo	102	25.8	21.5–30.4
	None	28	7.1	4.8–10.1
	Black	21	5.3	3.3–8.1
	Bantu	16	4.0	2.4–6.6
	Latin	15	3.8	2.2–6.3
	Others	214	54.0	48.9–59.0
If you have nationality (n = 628)	Yes	502	79.9	76.5–82.9
	No (stateless)	126	20.1	17.4–23.4
Last migratory status before coming to Brazil (n = 381)	National	208	54.6	49.4–59.6
	Migrant	87	22.8	18.7–27.4
	Irregular	38	10.0	7.2–13.5
	Refugee	14	3.7	2.1–6.2
	Others	34	8.9	6.3–12.3
If you did military service (n = 706)	Yes	42	5.9	4.3–8.0
	No	664	94.0	91.9–95.6
Manner of military service (n = 40)	Compulsory	31	77.5	61.1–88.6
	Volunteer	9	22.5	11.4–38.8
Means of transport used between the country of origin and Brazil (n = 781)	Air only	629	80.5	77.5–83.2
	Sea only	12	1.5	0.8–2.7
	Land only	78	10.0	8.0–12.3
	Air and sea	5	0.6	0.2–1.5
	Air and land	50	6.4	4.8–8.4
	Sea and land	2	0.3	0.0–1.0
	Air, sea and land	5	0.6	0.2–1.5
If you have any identity or travel documents (n = 716)	Yes	632	88.3	85.6–90.4
	No	84	11.7	9.5–14.3
Document used to enter Brazil (n = 701)	Passport	578	82.5	79.3–85.1
	Fake document	84	12.0	9.7–14.6
	Identity	19	2.7	1.6–4.2
	Visa	12	1.7	0.9–3.0
	Borrowed document	4	0.6	0.1–1.5
	No document	4	0.6	0.1–1.5

**Figure:**
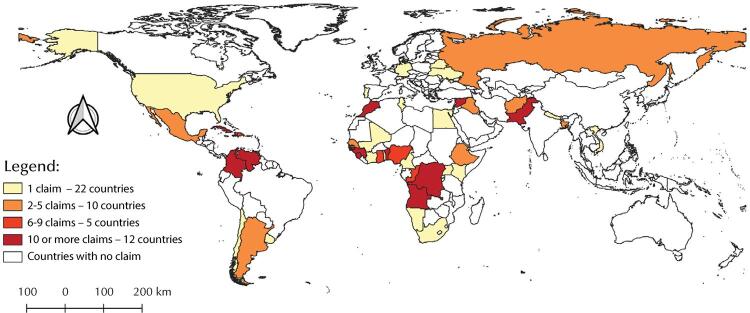
Asylum claims made at Cáritas Archdiocesana of Rio de Janeiro by country of birth, between 2016 and 2017 (818 requests by individuals from 49 different countries).

Among these claims, 510 (62.4%) were male, 797 (97.1%) were adults, with a mean age of 30.5 years (± 8.8), 551 (71.1%) were single, 216 (29.3%) had Spanish as their mother native language and 340 (44.1%) had higher education. Only 27 (4%) were unemployed in the country of origin before coming to Brazil. Five hundred fifteen (515, 80.3%) claimed to be Christians, and among these the majority were Catholics (223, 43.3%). One hundred twenty-six (126 – 20.1%) of them self-reported as stateless, and 38 (10%) had irregular migratory status before coming to Brazil (Table 1).

Among those who performed military service, 31 (77.5%) reported having done the military service compulsorily, having no option of choice. The journey to reach Brazil was carried out only by plane by 629 (80.5%) of asylum-seekers; 84 (11.7%) did not have identification or travel documents. To enter the country, 84 (12%) reported having used false documents, four (0.6%) had borrowed documents and four (0.6%) entered without any documents (Table 1).

As for the family situation, 101 (15.6%) said they were accompanied by children and adolescents in Brazil. Regarding international protection, four (0.5%) had previously been recognized as refugees in other countries, 89 (12.7%) had tried to obtain state protection in their country of origin, without success, and 65 (8.9%) returned to their country of origin after entering Brazil (Table 2).

**Table 2:** Information on family members and international protection for asylum-seekers assisted at Cáritas Arquidiocesana do Rio de Janeiro, between 2016 and 2017.

Family members and international protection	Yes (n)	%	95%CI
There are minors under 18 who accompany you in Brazil (n = 648)	101	15.6	12.9–18.6
Are you aware of a family member who is an asylum-seeker in Brazil? (n = 640)	96	15.0	12.3–18.0
Are you aware of a family member who has been recognized as a refugee in Brazil? (n = 638)	28	4.4	2.9–6.3
Are you aware of a family member who has been recognized as a refugee in another country? (n = 642)	17	2.6	1.5–4.2
Are you aware of any family members living in Brazil in a migratory situation other than refugee status? (n = 556)	64	11.5	9.0–14.5
Have you ever applied for asylum in Brazil (n = 767)	8	1.0	0.4–2.1
Have you already applied for asylum in another country (n = 696)	11	1.6	0.8–2.9
Have you been recognized as a refugee before (n = 792)	4	0.5	0.1–1.3
Sought state protection in country of origin or habitual residence (n = 727)	68	9.4	7.3–11.7
Internally moved within your country of origin or habitual residence in search of protection (n = 699)	89	12.7	10.3–15.4
If the applicant has returned to his/her country of origin or habitual residence after having entered Brazil (n = 731)	65	8.9	6.9–11.2

Regarding the situations experienced in the country of origin that motivated the asylum claim, 582 (84.7%) declared fear and risk of being a victim of torture; 345 (49.3%) a serious and generalized situation of violation of human rights, and 332 (47.4%) feared persecution due to political opinions (Table 3). Among the other reasons, economic issues, death threats, accusations of crimes and witchcraft, belonging to LGBTQI+ groups, and the search for access to health were declared.

**Table 3:** Reasons why forced migrants assisted at Cáritas Arquidiocesana do Rio de Janeiro apply for refugee status to the National Committee for Refugees, between 2016 and 2017.

Persecutions suffered	Yes (n)	%	95%CI
Due to race (n = 700)	55	7.9	6.0–10.1
Due to religion (n = 700)	68	9.7	7.6–12.2
Due to nationality (n = 700)	17	2.4	1.4–3.9
For being a member of a social group (n = 700)	93	13.3	10.9–16.0
Due to political opinion (n = 700)	332	47.4	43.6–51.2
Due to the serious and widespread violation of human rights in my country (n = 700)	345	49.3	45.5–53.0
Whether you could be a victim of torture or cruel, inhuman or degrading treatment if you return to your country of origin or habitual residence (n = 687)	582	84.7	81.7–87.2

Regarding health conditions, 216 (29.0%) of the applicants said they had some symptom, disease or condition, 49 (22.7%) felt pain in some part of the body, 28 (13%) reported vision problems, 14 (6.5%) reported having HIV or other infectious diseases, 14 (6.5%) reported having hypertension, and 20 (8.2%) of the women claimed to be pregnant at the time of completing the application. Only 15 migrants (2.2%) were undergoing medical or psychological treatment at the time of claim. Dental, ophthalmologic, obstetric, chemical dependency, arterial hypertension, diabetes and HIV/AIDS treatments were mentioned. Among the physical disabilities, hydrocephalus, arm paralysis and lower limb amputation were recorded. The visual impairments mentioned were mainly attributed to myopia; hearing impairments were not specified (Table 4).

**Table 4:** Health conditions self-reported by asylum-seekers assisted by Cáritas Arquidiocesana do Rio de Janeiro, total and five most frequent countries of birth, between 2016 and 2017.

Health profile	Yes (n)	%	95%CI
Do you have any symptoms, disease or aggravation? (n = 746)	216	29.0	25.7–32.3
Diseases or aggravations (n = 216):			
Pain in some part of the body	49	22.7	17.3–28.9
Vision problems	28	13.0	8.9–18.3
HIV or other infectious diseases	14	6.5	3.7–10.8
Hypertension	14	6.5	3.7–10.8
Skin conditions, allergies or itching	11	5.1	2.6–9.1
Respiratory diseases, including asthma	11	5.1	2.6–9.1
Digestive system diseases	11	5.1	2.6–9.1
Fractures or trauma from torture	6	2.8	1.1–6.2
Mental health disorders	5	2.3	0.8–5.6
Hearing diseases	4	1.9	0.5–4.9
Diabetes	4	1.9	0.5–4.9
Anemia	3	1.4	0.3–4.3
Unspecified fever	3	1.4	0.3–4.3
Other diseases and conditions	53	24.5	19.0–30.9
If you are a woman, are you pregnant? (n = 244)	20	8.2	5.2–12.5
If you undergo any medical or psychological treatment (n = 694)	15	2.2	1.2–3.6
Have a physical disability (n = 700)	14	2.0	1.1–3.4
Have a hearing impairment (n = 700)	4	0.6	0.1–1.5
Have a visual impairment (n = 700)	42	6.0	4.4–8.0

## DISCUSSION

As far as it was possible to identify, this was the first Brazilian study to analyze the sociodemographic and health profile of asylum-seekers. A previous study^12^described the profile only of those who received refugee status in the country but did not include questions about trajectory and health status. We found, in our study population, to be mostly composed by adult men, Christians, with higher education and who migrated using air transport. This sociodemographic profile coincides with that described for all refugees in Brazil^12^, which suggests that our sample is representative of the entire refugee population in our country. Indeed, Cáritas-RJ has become the main reference in the service and support to asylum-seekers and refugees, that is, a large part of this population residing in Rio de Janeiro, of different age groups, genders and socioeconomic situations, is concentrated there^11^.

This profile contrasts with that found in other countries, where most of asylum-seekers are children and adolescents or adults without higher education, who arrive by land and/or sea^6^. The profile found here suggests that a population with a higher socioeconomic level seeks Brazil, while the most disadvantaged seek countries neighboring the conflicts, or at least closer to them. Note that the period studied precedes the Venezuelan exodus and, therefore, does not extend to the population that arrived after 2018.

These people who fled persecution in their countries face adverse conditions when they arrive in Brazil, such as delays in obtaining refugee status, language difficulties, problems with documentation, and the presence of various symptoms, diseases and health problems, as we will discuss next. Contrary to the treatment given to Venezuelans, who benefited from rapid decision-making, less than 2% of applicants in this sample had received refugee status by the end of 2019, when we ended data collection.

Despite the high level of education, many tend to take on informal jobs, due to the difficulty of revalidating their diplomas^13^. Language is also a relevant barrier that generates social exclusion^14^. Completing the claims mainly in Portuguese denotes the action of interpreters, often Cáritas-RJ’s employees^11^. The importance of language in the migratory process possibly explains the high frequency of applicants born in Angola^15^. Portuguese courses for individuals from non-Portuguese-speaking countries can facilitate their entry into the job market in Brazil^14^. We were surprised by the strong presence of Cubans, as these immigrants usually go to the United States of America^16^. Although the *Mais Médicos* Program brought Cuban doctors to work in Brazil, none of the asylum-seekers had this profession. However, the social support networks created by forced migrants, including sheltering same nationality individuals in refugee situations, even if they do not know each other^17^, may explain the preference of non-medical Cubans for Brazil.

The use of false or borrowed documents, or even the lack of them, has already been identified as one of the main difficulties of reception in other countries^18^. Any foreigner must be able to prove that his presence in the country is legal^18^; in the case of asylum-seekers, the lack of documents can result in arrest or summary expulsion, with risk of life^18^.

A relevant result of this study concerns the reports of health and torture. As in other host countries, the reason for migration was mostly the experience or fear of torture and cruel treatment in the country of origin. Violence and mutilation are common in countries of origin^6^and can lead to serious mental health problems. In Denmark, 45% of asylum-seekers reported having been tortured and 40% of them were depressed and anxious compared to 10% who were not tortured; 63% met post-traumatic stress disorder criteria and 42% had related physical scars^19^.

The most self-reported health conditions in this study – non-communicable chronic diseases – corroborate reports recorded in other countries^20^. It should be noted that public health bodies in destination countries, as well as sectors of their populations, are mainly concerned with communicable diseases that would be brought by migrants, fueling xenophobic discourses in contrast to evidence pointing out that migrants tend to be vulnerable to diseases already existing in destination countries^21,22^. Access issues may explain this vulnerability: 20% of Sudanese refugees have never seen a dentist or ophthalmologist and 11% have never seen a doctor^23^. In Brazil, a country with the largest public health system in the world, the conditions reported in this study could be treated via access to public primary health care services.

This study has important limitations. First, it was based on secondary data, collected through an administrative form, not intended for research purposes. The information – self-reported – may have generated memory bias or self-censorship, for fear that refugee status would be denied. In addition, filling out the questionnaire soon after arriving in Brazil may have underestimated the prevalence of health conditions. A selection bias is also not ruled out, since a certain contingent of applicants may have dispensed with contacting Cáritas. Finally, the authors’ institutional agreements with Cáritas-RJ included dispensing with the collection of some more sensitive data, such as prison history.

In summary, asylum-seekers face difficulties in Brazil that can be mitigated with multisectoral government actions, such as stimulating demand for the SUS, assistance in documentation, and a greater offer of Portuguese language courses. It would be desirable to speed up the decision-making process on the asylum request, in order to facilitate the integration of these populations in Brazil and their inclusion in the health planning and management agenda in the three spheres of government.
