# Supplementary material for: Sociodemographic and health profile of asylum-seekers in Rio de Janeiro, 2016–2017
Source: Rev Saude Publica. 2022 Apr 13;56:31. doi: 10.11606/s1518-8787.2022056003956 (PMC9018066; doi:10.11606/s1518-8787.2022056003956)
Supplement: Translation

# Translation: Perfil sociodemográfico e de saúde de solicitantes de refúgio no Rio de Janeiro, 2016–2017

**Authors:** João Roberto Cavalcante, Raquel Proença, Ignacio Cano, Anete Trajman, Eduardo Faerstein

**Affiliations:** I Brasil Universidade do Estado do Rio de Janeiro. Instituto de Medicina Social. Rio de Janeiro, RJ, Brasil; II Brasil Universidade do Estado do Rio de Janeiro. Centro de Ciências Sociais. Rio de Janeiro, RJ, Brasil

**Keywords:** Refugiados, Características da População, Perfil de Saúde, Saúde Global

## Abstract

**OBJETIVO:**

Analisar o perfil sociodemográfico e as condições de saúde autorrelatadas por solicitantes de refúgio no Rio de Janeiro.

**MÉTODOS:**

Estudo transversal de dados secundários, coletados de formulários de solicitação de refúgio preenchidos em 2016 e 2017, na Cáritas Arquidiocesana do Rio de Janeiro (Cáritas-RJ). Foram realizadas análises descritivas e calculadas frequências absolutas, relativas e intervalos de 95% de confiança.

**RESULTADOS:**

Foram identificados formulários preenchidos por 818 solicitantes de refúgio, originários de 49 países diferentes, dos quais 126 (20,3%) eram apátridas, 510 (62,7%) do sexo masculino, 797 (97,4%) adultos, com idade média de 30,5 anos, 551 (73,5%) solteiros, 340 (44,1%) com ensino superior e 27 (4,0%) desempregados no país de origem antes da vinda para o Brasil. Entre os motivos declarados para solicitação de refúgio, destacaram-se o temor de perseguição por opinião política, violação de direitos humanos e risco de tortura. Para chegar ao Brasil, 629 (80,5%) viajaram somente de avião. Em relação às condições de saúde, 216 (29,0%) afirmaram ter algum sintoma, doença ou agravo em saúde, sendo as mais frequentes dores, problemas de visão, doenças infecciosas (incluindo HIV/aids) e hipertensão. Apenas 15 indivíduos (2,2%) relataram estar em algum tratamento médico ou psicológico; 42 (6,0%) relataram deficiências visuais, 14 (2,0%) relataram deficiências físicas e 4 (0,6%) deficiências auditivas.

**CONCLUSÕES:**

Diferentemente de outros países, onde migrantes forçados de baixo grau de instrução entram por vias clandestinas marítimas ou terrestres, os solicitantes de refúgio residentes no Rio de Janeiro entre 2016 e 2017, eram, em sua maioria, adultos com ensino superior que migraram usando transporte aéreo. Apresentavam condições de saúde sensíveis à atenção primária que poderiam ser tratadas via acesso aos serviços públicos da atenção primária em saúde.

## INTRODUÇÃO

Migrações forçadas não constituem um fenômeno histórico recente, pelo contrário, remontam à pré-História, quando há cerca de dois milhões de anos nossos ancestrais deslocaram-se da África para outras regiões do planeta, devido, principalmente, às questões climáticas e à escassez de alimentos^1^. Mais recentemente, outras migrações forçadas ocorreram por diferentes motivos, como conflitos e guerras, pobreza e fome, epidemias e busca por acesso a serviços de saúde^2^.

As migrações forçadas classificam-se nas seguintes subcategorias: refugiados, solicitantes de refúgio e deslocados internos. Refugiados são indivíduos que receberam status de proteção do país para onde migraram, por possuírem fundado temor de perseguição por raça, religião, nacionalidade, grupo social, opiniões políticas ou situação de grave e generalizada violação de direitos humanos^3^, encontram-se fora do país de sua nacionalidade e não podem ou, em virtude desse temor, não querem valer-se da proteção desse país^4^. Solicitantes de refúgio são indivíduos que já realizaram migração internacional e pretendem ser admitidos no país de destino como refugiados e estão aguardando a decisão sobre o seu pedido de refúgio nos termos dos instrumentos internacionais e nacionais pertinentes^5^. Deslocados internos são indivíduos com razões de migração semelhantes às dos refugiados e solicitantes de refúgio, mas que não atravessaram uma fronteira internacional^6^.

Atualmente ocorre no mundo a maior onda de migrações forçadas desde a Segunda Guerra Mundial. Segundo o alto comissariado das nações unidas para refugiados (ACNUR), em 2019 existiam 79,5 milhões de migrantes forçados no mundo (1% da população mundial): 26 milhões de refugiados, 4,2 milhões de solicitantes de refúgio, 45,7 milhões de deslocados internos e 3,6 milhões de venezuelanos deslocados para o exterior^6^.

No Brasil, em 2019, havia 43 mil indivíduos com status de refugiados reconhecidos nos 10 anos anteriores, vindos de 90 países diferentes, dos quais 90% da Venezuela^7^. Naquele ano houve 82.500 novas solicitações de refúgio. O Comitê Nacional para Refugiados (CONARE) reconheceu 38 mil venezuelanos como refugiados entre dezembro de 2019 e abril de 2020 (cinco meses), porém milhares de solicitações realizadas antes e após esse período ainda estão pendentes e acumuladas aguardando decisão^6^.

A migração pode ter impacto negativo direto nas condições de saúde dos indivíduos. Doenças e agravos como tuberculose, sífilis, hipertensão, diabetes mellitus, obesidade, depressão, ansiedade, transtorno do estresse pós-traumático, sequelas de torturas, mutilações (incluindo mutilações genitais femininas) e violência sexual são frequentes nessa população^8,9^. As principais dificuldades enfrentadas após a chegada ao país de destino incluem os trabalhos precários, incompreensão da sua cultura, dificuldades de comunicação e dificuldades no acesso à saúde^8^. O acesso aos serviços de saúde e à garantia de moradia, trabalho e renda são exemplos de políticas públicas que podem mitigar os efeitos adversos das experiências pós-migração^10^.

O Brasil vem sendo buscado como país acolhedor por um número crescente de migrantes forçados. Pouco se conhece sobre o perfil e a saúde dessa população. No Rio de Janeiro, a Cáritas Arquidiocesana (Cáritas-RJ) presta assistência a essa população, por exemplo auxiliando-a nas tratativas de solicitação de refúgio e facilitando seu acesso ao Sistema Único de Saúde (SUS). A Cáritas-RJ é uma organização vinculada à Igreja católica, criada em 1976, quando o então arcebispo do Rio de Janeiro, Dom Eugênio Sales, iniciou um trabalho pioneiro de assistência a perseguidos políticos das ditaduras que chegavam ao Rio de Janeiro vindos de países vizinhos, como Argentina, Chile e Uruguai^11^.

A Cáritas-RJ segue acolhendo todos os anos centenas de refugiados e solicitantes de refúgio e mantém arquivados em papel os formulários de solicitação de refúgio que contêm diversos dados sobre essa população^11^. Até onde se sabe, não se conduziram estudos baseados nesses dados. Este estudo teve o objetivo de analisar o perfil sociodemográfico e as condições de saúde autorrelatadas por solicitantes de refúgio no Rio de Janeiro atendidos nessa instituição.

## MÉTODOS

Foi realizado um estudo transversal com coleta de dados secundários retrospectivos, cuja população foram os indivíduos que preencheram o formulário de solicitação de refúgio do CONARE na Cáritas-RJ em 2016 e 2017, quando o formulário passou a conter informações sobre saúde. Adicionalmente, foram coletadas informações que constam em entrevistas realizadas pela equipe de assistentes sociais da Cáritas-RJ.

Na Cáritas-RJ, os solicitantes de refúgio tinham a opção de responder o formulário em português, espanhol, inglês e francês. Quando o solicitante não dominava alguma dessas quatro línguas, sendo falante, por exemplo, de kikongo, lingala, árabe etc., intérpretes ajudavam no preenchimento do formulário. Os formulários do período do estudo possuíam 26 páginas com 103 perguntas e os dados foram extraídos por 12 digitadores entre abril de 2018 e dezembro de 2019.

Foi construída uma máscara do formulário para extração de dados utilizando o software EpiData 4.2.0.0. Os 12 digitadores foram treinados para realizar a coleta dos dados dos formulários de solicitação de refúgio e manejo do EpiData. Três pré-testes da máscara foram realizados pelos 12 digitadores, que extraíram dados de uma amostra de 20 formulários, com a finalidade de comparar e homogeneizar as digitações e fazer adequações à máscara. Os formulários foram classificados por língua antes do início da coleta, para que cada digitador coletasse os formulários das línguas em que fosse fluente. Semanalmente, dois supervisores verificavam cerca de 10% dos formulários digitados, a fim de detectar e corrigir possíveis erros.

Foram realizadas análises descritivas das variáveis categóricas e calculadas as frequências absolutas e relativas com seus respectivos intervalos de 95% de confiança (IC95%). As variáveis de respostas abertas, isto é, que não eram múltipla escolha, foram apresentadas com as respostas mais frequentes. Essas mesmas análises foram repetidas separadamente para solicitantes de refúgio dos cinco países de origem mais frequentes. Os dados faltantes não foram incluídos nos cálculos das frequências. Também foi elaborado um mapa temático com o número de solicitações de refúgio por país de origem. As análises de dados foram realizadas no software R 3.4.2 e o mapa temático foi elaborado no software QGIS 3.14.16.

O projeto foi aprovado pelo comitê de ética em pesquisa do Instituto de Medicina Social da Universidade do Estado do Rio de Janeiro (IMS/UERJ) sob o parecer 2.437.258.

## RESULTADOS

Foram identificados e incluídos 818 formulários de solicitações de refúgio referentes ao período do estudo, dos quais 529 (64,7%) de 2016 e 289 (35,3%) de 2017. Desses, apenas 11 (1,3%) tiveram o pedido de refúgio aceito até o momento da coleta (Tabela 1). Foram identificados 49 países de nascimento. Os 12 países que possuíam mais de 10 solicitações de refúgio foram: Angola (231 solicitações, 28,2% do total), República Democrática do Congo (153, 18,7%), Venezuela (133, 16,2%), Cuba (69, 8,4%), Síria (34, 4,1%), Guiné-Bissau (20, 2,4%), Guiné (18, 2,2%), Paquistão (16, 1,9%), Colômbia (15, 1,8%), Serra Leoa (15, 1,8%), Benim (12, 1,4%) e Marrocos (12, 1,4%) (Figura).

**Tabela 1:** Perfil sociodemográfico de solicitantes de refúgio atendidos na Cáritas Arquidiocesana do Rio de Janeiro, entre 2016 e 2017.

Características sociodemográficas	Respostas	n	%	IC95%
Ano de preenchimento do formulário (n = 818)	2016	529	64,7	61,2–67,9
	2017	289	35,3	32,0–38,7
*Status* da solicitação de refúgio (n = 818)	Pendente	758	92,7	90,6–94,3
	Aceito	11	1,3	0,7–2,4
	Recusado	1	0,1	0,0–0,7
	Não informado	48	5,9	4,4–7,7
Sexo (n = 818)	Masculino	510	62,4	58,9–68,6
	Feminino	308	37,6	34,3–41,0
Faixa etária em anos (n = 818)	Menor de 18 anos	21	2,6	1,6–3,9
	18 a 29 anos	427	52,2	48,7–55,6
	30 a 44 anos	315	38,5	35,1–41,9
	45 a 59 anos	52	6,4	4,8–8,3
	60 anos ou mais	3	0,4	0,0–1,1
Idioma do formulário (n = 818)	Português	292	35,7	32,4–39,1
	Espanhol	233	28,5	25,4–31,7
	Francês	206	25,2	22,2–28,3
	Inglês	87	10,6	8,6–13,0
Língua materna (n = 737)	Espanhol	216	29,3	26,0–32,7
	Português	185	25,1	22,0–28,4
	Lingala	83	11,3	9,1–13,8
	Kikongo	41	5,6	4,0–7,5
	Árabe	37	5,0	3,6–6,9
	Outras	175	23,7	20,7–27,0
Segundo idioma (n = 471)	Inglês	105	22,3	18,6–26,3
	Francês	99	21,0	17,4–25,0
	Português	91	19,3	15,9–23,2
	Lingala	46	9,8	7,3–12,8
	Kikongo	37	7,9	5,6–10,7
	Outros	93	19,7	16,3–23,6
Estado Civil (n=775)	Solteiro	551	71,1	67,7–74,2
	Casado	199	25,7	22,6–28,9
	Divorciado	11	1,4	0,7–2,6
	Separado/Desquitado	5	0,6	0,2–1,5
	Viúvo	4	0,5	0,1–1,4
	União Estável	2	0,3	0,0–1,0
	União Livre	3	0,4	0,1–1,2
Escolaridade (n = 771)	Superior	340	44,1	40,5–47,7
	Médio	312	40,5	36,9–44,0
	Fundamental	119	15,4	12,9–18,2
Setor da ocupação no país de origem (n = 675)	Serviços	348	51,5	47,7–55,4
	Comércio	99	15,6	12,1–17,6
	Indústria	65	9,6	7,5–12,1
	Agrária e agropecuária	4	0,6	0,1–1,6
	Desempregados	27	4,0	2,7–5,8
	Outros	132	19,5	16,6–22,7
Religião (n=641)	Cristianismo	515	80,3	77,0–83,3
	Islamismo	71	11,1	8,8–13,8
	Sem religião	25	3,9	2,6–5,7
	Ateu	3	0,5	0,1–1,4
	Outras	27	4,2	2,8–6,1
Denominações Cristãs (n = 515)	Católicos	223	43,3	38,9–47,7
	Pentecostais	76	14,8	11,8–18,1
	Testemunhas de Jeová	21	4,1	2,6–6,2
	Adventistas do 7° Dia	8	1,6	0,7–3,1
	Protestantes	6	1,2	0,4–2,6
	Cristãos – sem especificação	181	35,1	31,0–39,4
Etnia (n = 396)	Bakongo	102	25,8	21,5–30,4
	Nenhuma	28	7,1	4,8–10,1
	Negra	21	5,3	3,3–8,1
	Bantu	16	4,0	2,4–6,6
	Latina	15	3,8	2,2–6,3
	Outras	214	54,0	48,9–59,0
Se tem nacionalidade (n = 628)	Sim	502	79,9	76,5–82,9
	Não (apátrida)	126	20,1	17,4–23,4
Último status migratório antes de vir para o Brasil (n = 381)	Nacional	208	54,6	49,4–59,6
	Migrante	87	22,8	18,7–27,4
	Irregular	38	10,0	7,2–13,5
	Refugiado	14	3,7	2,1–6,2
	Outros	34	8,9	6,3–12,3
Se prestou serviço militar (n = 706)	Sim	42	5,9	4,3–8,0
	Não	664	94,0	91,9–95,6
Maneira que prestou serviço militar (n = 40)	Compulsória	31	77,5	61,1–88,6
	Voluntária	9	22,5	11,4–38,8
Meios de transportes usados entre o país de origem e o Brasil (n = 781)	Somente aéreo	629	80,5	77,5–83,2
	Somente marítimo	12	1,5	0,8–2,7
	Somente terrestre	78	10,0	8,0–12,3
	Aéreo e marítimo	5	0,6	0,2–1,5
	Aéreo e terrestre	50	6,4	4,8–8,4
	Marítimo e terrestre	2	0,3	0,0–1,0
	Aéreo, marítimo e terrestre	5	0,6	0,2–1,5
Se tem algum documento de identidade ou de viagem (n = 716)	Sim	632	88,3	85,6–90,4
	Não	84	11,7	9,5–14,3
Documento que usou para entrar no Brasil (n = 701)	Passaporte	578	82,5	79,3–85,1
	Documento falso	84	12,0	9,7–14,6
	Identidade	19	2,7	1,6–4,2
	Visto	12	1,7	0,9–3,0
	Documento emprestado	4	0,6	0,1–1,5
	Nenhum documento	4	0,6	0,1–1,5

**Figura:** Solicitações de refúgio realizadas na Cáritas Arquidiocesana do Rio de Janeiro por país de nascimento, entre 2016 e 2017 (818 solicitações de indivíduos de 49 países diferentes).

Entre esses solicitantes, 510 (62,4%) eram do sexo masculino, 797 (97,1%) adultos, com idade média de 30,5 anos (± 8,8), 551 (71,1%) eram solteiros, 216 (29,3%) tinham o espanhol como língua materna e 340 (44,1%) possuíam ensino superior. Apenas 27 (4%) estavam desempregados no país de origem antes da vinda para o Brasil. Afirmaram ser cristãos 515 (80,3%), sendo que entre esses a maioria era de católicos (223, 43,3%). Também foram identificados 126 (20,1%) apátridas e 38 (10%) estavam com o status migratório irregular antes de vir para o Brasil (Tabela 1).

Entre os que prestaram serviço militar, 31 (77,5%) relataram ter prestado o serviço militar de maneira compulsória, não tendo opção de escolha. A trajetória para chegar ao Brasil foi realizada somente de avião por 629 (80,5%) dos solicitantes de refúgio; 84 (11,7%) não possuíam documentos de identificação ou de viagem. Para entrar no país, 84 (12%) declararam ter utilizado documento falso, quatro (0,6%) portavam documento emprestado e quatro (0,6%) entraram sem nenhum documento (Tabela 1).

Quanto à situação familiar, 101 (15,6%) afirmaram estarem acompanhados de crianças e adolescentes no Brasil. Em relação à proteção internacional, quatro (0,5%) tinham sido reconhecidos previamente como refugiados em outros países, 89 (12,7%) tentaram conseguir proteção estatal no seu país de origem, sem sucesso, e 65 (8,9%) retornaram ao país de origem após ter entrado no Brasil (Tabela 2).

**Tabela 2:** Informações sobre familiares e proteção internacional aos solicitantes de refúgio atendidos na Cáritas Arquidiocesana do Rio de Janeiro, entre 2016 e 2017.

Familiares e proteção internacional	Sim (n)	%	IC95%
Existem menores de 18 anos que o (a) acompanham no Brasil (n = 648)	101	15,6	12,9–18,6
Tem conhecimento de algum familiar que é solicitante de refúgio no Brasil (n = 640)	96	15,0	12,3–18,0
Tem conhecimento de algum familiar que foi reconhecido como refugiado no Brasil (n = 638)	28	4,4	2,9–6,3
Tem conhecimento de algum familiar que foi reconhecido como refugiado em outro país (n = 642)	17	2,6	1,5–4,2
Tem conhecimento de algum familiar que vive no Brasil em outra situação migratória, que não a de refugiado (n = 556)	64	11,5	9,0–14,5
Já solicitou refúgio no Brasil (n = 767)	8	1,0	0,4–2,1
Já solicitou refúgio em outro país (n = 696)	11	1,6	0,8–2,9
Já foi reconhecido como refugiado antes (n = 792)	4	0,5	0,1–1,3
Buscou proteção estatal no país de origem ou residência habitual (n = 727)	68	9,4	7,3–11,7
Deslocou-se internamente dentro do seu país de origem ou residência habitual em busca de proteção (n = 699)	89	12,7	10,3–15,4
Se o solicitante retornou ao seu país de origem ou residência habitual depois de ter entrado no Brasil (n = 731)	65	8,9	6,9–11,2

Sobre as situações vividas no país de origem que motivaram a solicitação de refúgio, 582 (84,7%) declararam temor e risco de ser vítima de tortura; 345 (49,3%), situação grave e generalizada de violação de direitos humanos, e 332 (47,4%) temor de perseguição por opinião política (Tabela 3). Entre os demais motivos, foram declaradas questões econômicas, ameaças de morte, acusações de crimes e feitiçarias, pertencer a grupos LGBTQI+ e busca por acesso à saúde.

**Tabela 3:** Motivos pelos quais o migrante forçado atendido na Cáritas Arquidiocesana do Rio de Janeiro solicita status de refugiado ao Comitê Nacional para Refugiados, entre 2016 e 2017.

Perseguições sofridas	Sim (n)	%	IC95%
Por raça (n = 700)	55	7,9	6,0–10,1
Por religião (n = 700)	68	9,7	7,6–12,2
Por nacionalidade (n = 700)	17	2,4	1,4–3,9
Por ser membro de um grupo social (n = 700)	93	13,3	10,9–16,0
Por opinião política (n = 700)	332	47,4	43,6–51,2
Por situação de grave e generalizada violação de direitos humanos no meu país (n = 700)	345	49,3	45,5–53,0
Se poderá ser vítima de tortura ou tratamento cruel, desumano ou degradante caso retorne ao seu país de origem ou residência habitual (n = 687)	582	84,7	81,7–87,2

Em relação às condições de saúde, 216 (29,0%) dos solicitantes afirmaram ter algum sintoma, doença ou agravo, sendo que 49 (22,7%) sentiam dores em alguma parte do corpo, 28 (13%) relataram problemas de visão, 14 (6,5%) acusaram ter HIV ou outras doenças infecciosas, 14 (6,5%) declararam ter hipertensão e 20 (8,2%) das mulheres afirmaram estar grávidas no momento de preenchimento do formulário. Apenas 15 migrantes (2,2%) faziam algum tratamento médico ou psicológico no momento de preenchimento do formulário. Foram mencionados tratamentos odontológico, oftalmológico, obstétrico, para dependência química, para hipertensão arterial, para diabetes e HIV/aids. Entre as deficiências físicas, foram registradas hidrocefalia, paralisia do braço e amputação de membro inferior. As deficiências visuais mencionadas foram atribuídas principalmente à miopia; deficiências auditivas não foram especificadas (Tabela 4).

**Tabela 4:** Condições de saúde autorrelatadas pelos solicitantes de refúgio atendidos pela Cáritas Arquidiocesana do Rio de Janeiro, total e cinco países de nascimento mais frequentes, entre 2016 e 2017.

Perfil de saúde	Sim (n)	%	IC95%
Possui algum sintoma, doença ou agravo? (n = 746)	216	29,0	25,7–32,3
Doenças ou agravos (n = 216):			
Dor em alguma parte do corpo	49	22,7	17,3–28,9
Problemas de visão	28	13,0	8,9–18,3
HIV ou outras doenças infecciosas	14	6,5	3,7–10,8
Hipertensão	14	6,5	3,7–10,8
Doenças de pele, alergias ou prurido	11	5,1	2,6–9,1
Doenças respiratórias, inclusive asma	11	5,1	2,6–9,1
Doenças do sistema digestivo	11	5,1	2,6–9,1
Fraturas ou traumas por tortura	6	2,8	1,1–6,2
Transtornos de saúde mental	5	2,3	0,8–5,6
Doenças do aparelho auditivo	4	1,9	0,5–4,9
Diabetes	4	1,9	0,5–4,9
Anemia	3	1,4	0,3–4,3
Febre não especificada	3	1,4	0,3–4,3
Outras doenças e agravos	53	24,5	19,0–30,9
Caso seja mulher, está grávida? (n = 244)	20	8,2	5,2–12,5
Se faz algum tratamento médico ou psicológico (n = 694)	15	2,2	1,2–3,6
Possui alguma deficiência física (n = 700)	14	2,0	1,1–3,4
Possui alguma deficiência auditiva (n = 700)	4	0,6	0,1–1,5
Possui alguma deficiência visual (n = 700)	42	6,0	4,4–8,0

## DISCUSSÃO

Até onde foi possível identificar, este foi o primeiro estudo brasileiro a analisar o perfil sociodemográfico, bem como de saúde, de solicitantes de refúgio. Um estudo anterior^12^ descreveu o perfil apenas dos que receberam o status de refugiados no país, mas não incluiu perguntas sobre trajetória e condições de saúde. Encontramos, em nossa população de estudo, uma maioria de homens adultos, cristãos, com ensino superior e que migraram usando meio de transporte aéreo. Esse perfil sociodemográfico coincide com o descrito para a totalidade dos refugiados do Brasil^12^. Isso sugere que nossa amostra é representativa do conjunto da população refugiada no nosso país. Com efeito, a Cáritas-RJ tornou-se a principal referência no atendimento e apoio aos solicitantes de refúgio e refugiados, ou seja, ali concentra-se grande parte dessa população residente do Rio de Janeiro, de diferentes faixas etárias, gêneros e situações socioeconômicas^11^.

Esse perfil contrasta com o encontrado em outros países, onde a maioria de requerentes de asilo é composta por crianças e adolescentes ou adultos sem ensino superior, que chegam por via terrestre e/ou marítima^6^. O perfil aqui encontrado sugere que uma população com nível socioeconômico mais elevado procura o Brasil, enquanto os mais desfavorecidos procuram países vizinhos aos conflitos ou, pelo menos, mais próximos. Note-se que o período estudado precede ao êxodo venezuelano e, portanto, não se estende à população que chegou após 2018.

Essas pessoas que fugiram da perseguição em seus países enfrentam condições adversas ao chegarem ao Brasil, como demora na obtenção do status de refugiado, dificuldade com a língua, problemas com documentação e a presença de diversos sintomas, doenças e agravos de saúde, como discutiremos a seguir. Ao contrário do tratamento dado aos venezuelanos, que se beneficiaram de grande celeridade na decisão, menos de 2% dos solicitantes dessa amostra recebera status de refugiado até o final de 2019, quando encerramos a coleta de dados.

Apesar do elevado grau de instrução, muitos costumam assumir empregos informais, devido à dificuldade de revalidação dos seus diplomas^13^. O idioma também constitui uma barreira relevante que gera exclusão social^14^. O preenchimento do formulário principalmente em português denota a ação de intérpretes, muitas vezes funcionários da Cáritas-RJ^11^. A importância do idioma no processo migratório possivelmente explica a grande frequência de solicitantes nascidos em Angola^15^. Cursos de português para indivíduos de países não lusófonos podem facilitar sua entrada no mercado de trabalho no Brasil^14^. Surpreendeu-nos a forte presença de cubanos, pois esses imigrantes costumam ir para os Estados Unidos da América^16^. Embora o Programa Mais Médicos tenha trazido médicos cubanos para atuar no Brasil, nenhum dos solicitantes de refúgio tinha essa profissão. No entanto, as redes sociais de apoio que migrantes forçados criam, inclusive com acolhimento em suas residências de indivíduos em situação de refúgio da mesma nacionalidade, ainda que não se conheçam^17^, pode explicar a preferência de cubanos não médicos pelo Brasil.

A utilização de documentos falsos ou emprestados ou mesmo sua falta, já foi apontada como uma das principais dificuldades de acolhimento em outros países^18^. Qualquer estrangeiro deve ser capaz de provar que sua presença no país é legal^18^; no caso de solicitantes de refúgio, a falta de documentos pode resultar na detenção ou expulsão sumária, com risco de vida^18^.

Um resultado relevante deste estudo diz respeito aos relatos de saúde e tortura. Assim como em outros países de acolhimento, o motivo para a migração foi, em sua maioria, a experiência ou o temor da tortura e tratamento cruel no país de origem. Violências e mutilações são comuns nos países de origem^6^ e podem gerar sérios problemas de saúde mental. Na Dinamarca, 45% dos solicitantes de refúgio declararam ter sido torturados e 40% deles estavam deprimidos e ansiosos em comparação aos 10% que não foram torturados; 63% preenchiam os critérios de transtorno de estresse pós-traumático e 42% tinham cicatrizes físicas relacionadas^19^.

Finalmente, embora infrequentes, as condições de saúde mais autorrelatadas neste estudo – doenças crônicas não transmissíveis – corroboram relatos registrados em outros países^20^. Note-se que órgãos de saúde pública em países de destino, assim como setores de suas populações, preocupam-se principalmente com doenças transmissíveis que seriam trazidas pelos migrantes, alimentando discursos xenofóbicos em contraste com evidências apontando que migrantes tendem a ser vulneráveis a doenças já existentes nos países de destino^21,22^. Questões de acesso podem explicar essa vulnerabilidade: 20% dos refugiados sudaneses nunca consultaram um dentista ou oftalmologista e 11% nunca consultaram um médico^23^. No Brasil, país com o maior sistema público de saúde do mundo, as condições relatadas neste estudo poderiam ser tratadas via acesso aos serviços públicos da atenção primária em saúde.

Este estudo tem limitações importantes. Primeiramente, o estudo se baseou em dados secundários, coletados por meio de um formulário com caráter administrativo, não planejado para fins de pesquisa. As informações – autorrelatadas – podem ter gerado viés de memória ou autocensura, por temor de que o status de refúgio fosse negado. Além disso, o preenchimento logo após a chegada ao Brasil pode ter subestimado a prevalência de condições de saúde. Também não está descartado algum viés de seleção, já que um certo contingente de solicitantes pode ter dispensado o contato com a Cáritas. Finalmente, os acordos institucionais dos autores com a Cáritas-RJ incluíram dispensar a coleta de alguns dados mais sensíveis, como histórico de prisão.

Em resumo, os solicitantes de refúgio enfrentam dificuldades no Brasil que podem ser atenuadas com ações governamentais multissetoriais, como o estímulo à procura do SUS, auxílio na documentação e maior oferta de cursos de Língua Portuguesa. Seria desejável maior celeridade no processo de decisão sobre a solicitação de refúgio, a fim de facilitar a integração dessas populações no Brasil e a sua inclusão na agenda de planejamento e gestão em saúde nas três esferas de governo.
